# Mitochondrial Transport from Mesenchymal Stromal Cells to Chondrocytes Increases DNA Content and Proteoglycan Deposition *In Vitro* in 3D Cultures

**DOI:** 10.1177/19476035221126346

**Published:** 2022-10-19

**Authors:** Jasmijn V. Korpershoek, Margot Rikkers, Fleur S. A. Wallis, Koen Dijkstra, Marije te Raa, Peter de Knijff, Daniel B. F. Saris, Lucienne A. Vonk

**Affiliations:** 1 UMC Utrecht, Utrecht, The Netherlands; 2 Leids Universitair Medisch Centrum, Leiden, The Netherlands; 3 CO.DON AG, Teltow, Germany

**Keywords:** cartilage repair, repair, mesenchymal stem cells, cells, articular cartilage, tissue, mitochondria, cell communication

## Abstract

**Objective:**

Allogeneic mesenchymal stromal cells (MSCs) are used in the 1-stage treatment of articular cartilage defects. The aim of this study is to investigate whether transport of mitochondria exists between chondrocytes and MSCs and to investigate whether the transfer of mitochondria to chondrocytes contributes to the mechanism of action of MSCs.

**Design:**

Chondrocytes and MSCs were stained with MitoTracker, and CellTrace was used to distinguish between cell types. The uptake of fluorescent mitochondria was measured in cocultures using flow cytometry. Transport was visualized using fluorescence microscopy. Microvesicles were isolated and the presence of mitochondria was assessed. Mitochondria were isolated from MSCs and transferred to chondrocytes using MitoCeption. Pellets of chondrocytes, chondrocytes with transferred MSC mitochondria, and cocultures were cultured for 28 days. DNA content and proteoglycan content were measured. Mitochondrial DNA of cultured pellets and of repair cartilage tissue was quantified.

**Results:**

Mitochondrial transfer occurred bidirectionally within the first 4 hours until 16 hours of coculture. Transport took place via tunneling nanotubes, direct cell-cell contact, and extracellular vesicles. After 28 days of pellet culture, DNA content and proteoglycan deposition were higher in chondrocyte pellets to which MSC mitochondria were transferred than the control groups. No donor mitochondrial DNA was traceable in the biopsies, whereas an increase in MSC mitochondrial DNA was seen in the pellets.

**Conclusions:**

These results suggest that mitochondrial transport plays a role in the chondroinductive effect of MSCs on chondrocytes *in vitro.* However, *in vivo* no transferred mitochondria could be traced back after 1 year.

## Introduction

Multipotent mesenchymal stromal (stem) cells (MSCs) can be isolated from bone marrow, adipose tissue, synovial membrane, and other tissues.^1^ Due to their multilineage differentiation potential,^2^ anti-inflammatory properties,^3^ and signaling through trophic factors^4^ and extracellular vesicles (EV),^5^ MSCs are used in a wide spectrum of regenerative treatments. One of the treatments employing MSCs is IMPACT (Instant MSC Product accompanying Autologous Chondron Transplantation). IMPACT is a new treatment for articular cartilage defects of the knee and combines 10% recycled autologous chondrons with 90% off-the-shelf available allogeneic MSCs.^6-8^ Results of a phase I/II trial using IMPACT for treatment of articular cartilage defects showed safety and feasibility of this procedure,^6,7^ and 5-year clinical outcomes were promising.^8^ The repaired cartilage defect site did not contain autosomal DNA of the MSC donors, suggesting that the MSCs do not differentiate, but rather act as signaling cells,^9,10^ possibly through secretion of chondroinductive^11,12^ and anti-inflammatory agents.^13^

The transfer of organelles, such as mitochondria, might also contribute to the stimulatory effect of MSCs on chondrogenesis. MSC-derived mitochondria enhanced the phagocytic capacity of alveolar macrophages and ameliorated lung injury by improving mitochondrial function and adenosine triphosphate (ATP) turnover in a murine model.^14,15^ Furthermore, transplanted MSC mitochondria restored mitochondrial function and decreased apoptosis in rabbit cardiomyocytes postischemia,^16^ and intramyocardial injection of autologous mitochondria improved ventricular function in patients with ischemic injury.^17^ While the occurrence of mitochondrial transfer from equine, mice, and rat MSCs toward chondrocytes (CH) has been described,^18-20^ it has not been demonstrated in human cells before. Moreover, it is unclear whether transport takes place from CH to MSC as well. As shown in other tissues than cartilage, transfer of mitochondria can play a role in tissue repair, but its role in MSC-stimulated chondrogenesis is unknown. CH need ATP for the production of the main components of cartilage glycosaminoglycans (GAGs) and type II collagen,^21^ which is provided normally by anaerobic glycolysis.^22^ Under glucose-deprived conditions or glycolysis inhibition, CH switch to oxidative phosphorylation to maintain ATP production.^23^ Thus, the presence of functional mitochondria in CH is of paramount importance for their prolonged survival. Mitochondrial dysfunction can develop after pathological mechanical loading^24^ and is one of the hallmarks in the development of osteoarthritis.^25^ Transfer of functional mitochondria could prevent or resolve this mitochondrial dysfunction. Therefore, the aim of this study is to investigate whether mitochondrial transfer takes place between human CH and MSCs. We study the timing of mitochondrial transfer as well as different modes of transport *in vitro*. In addition, we investigate the effect of inflammation and senescence on mitochondrial transfer by preincubating with tumor necrosis factor α (TNF-α) and mitomycin C. Using MitoCeption,^26^ we analyze the effect of transferring MSC-derived mitochondria to CH on DNA content and proteoglycan deposition in 3-dimensional (3D) cultures. Finally, to study mitochondrial transfer *in vivo*, we isolate DNA from cartilage biopsies of 6 patients treated with IMPACT^6,7^ and use single-nucleotide polymorphism (SNP) genotyping to determine the presence of MSC donor mitochondrial DNA.

## Methods

### Donors and Cell Isolation

Human MSCs were isolated from the bone marrow of healthy donors in the GMP-licensed Cell Therapy Facility (Department of Clinical Pharmacy, University Medical Center Utrecht) as approved by the Dutch Central Committee on Research Involving Human Subjects (CCMO, Bio-banking bone marrow for MSC expansion, NL41015.041.12). The parent or legal guardian of the donor signed the informed consent approved by the CCMO (*n* = 5, age range = 2-12). In brief, the mononuclear fraction was separated and MSCs were isolated by plastic adherence and expanded for 3 passages in Minimum Essential Media (αMEM; Macopharma, Utrecht, The Netherlands) with 5% (v/v) platelet lysate and 3.3 IU/ml heparin and cryopreserved. Subsequently, MSCs were culture-expanded for 2 or 3 additional passages in MSC expansion medium, αMEM (Gibco, Bleijswijk, The Netherlands), 10% (v/v) fetal bovine serum (FBS; Biowest, Nuaillé, France), 1% penicillin/streptomycin (pen/strep; 100 U/ml/100 µg/ml; Gibco), 200 µM l-ascorbic acid 2-phosphate (ASAP; Sigma-Aldrich, Saint-Louis, MO), and 1 ng/ml basic fibroblast growth factor (bFGF; PeproTech, London, UK).

Cartilage was obtained after debridement of focal cartilage lesions from patients undergoing autologous chondrocyte implantation (ACI) and is considered medical waste or redundant material (*n* = 5, age range = 18-38). The tissue collection was performed according to the Medical Ethics regulations of the University Medical Center Utrecht and the guideline “Human Tissue and Medical Research: Code of Conduct for responsible use” of the Dutch Federation of Medical Research Societies.^27,28^ CH were isolated from the debrided cartilage by digestion of 0.2% (w/v) pronase (Sigma-Aldrich) in Dulbecco’s Modified Eagle Medium (DMEM, 31966; Gibco) with 1% pen/strep for 2 hours, followed by overnight digestion of 0.075% (w/v) collagenase II (CLS-2; Worthington, Lakewood, NJ) in DMEM supplemented with 10% FBS and 1% pen/strep. Isolated CH were culture-expanded to passage 2 in chondrocyte expansion medium (DMEM, 10% FBS, 1% pen/strep).

### Quantification of Monolayer Mitochondrial Transfer

To enable identification of the different donor and receiving cell type in culture, the donor cell type was labeled with CellTrace Violet (Invitrogen, Carlsbad, CA) and MitoTracker Red CMXRos (Molecular Probes, Invitrogen) according to the manufacturer’s instructions. Receiving cells were unlabelled. Cells were stained 1 day prior to initiation of the coculture. In addition, cells were pretreated with 0.02 µg/ml mitomycin C (Substipharm, Paris, France) for 6 days to induce senescence^29^ or with 10 ng/ml TNF-α (R&D Systems, Minneapolis, MN) for 24 hours to mimic an *in vitro* inflammatory environment.^30^

MSCs (passage 5 or 6) and CH (passage 2) were seeded in 6-well plates at a density of 100,000 cells per well in a 1:1 ratio. Dual-stained donor cells were plated 24 hours before initiation of the coculture. Unstained receiving cells were added to the preseeded donor cells and cocultures were maintained for 24 hours in chondrocyte expansion medium. After 0, 4, 8, 16, and 24 hours, cocultures were trypsinized, washed, and resuspended in phosphate-buffered saline (PBS) supplemented with 0.4% (v/v) human serum albumin (HSA; Albuman, Sanquin, Amsterdam, The Netherlands). Samples were analyzed using a CytoFLEX S flow cytometer (Beckman Coulter, Brea, CA). For each condition, 20,000 events were recorded. Flow cytometry results were extracted and analyzed using RStudio (R Core Team, Vienna, Austria) and FlowJo V10 data analysis software package (Tree Star Inc, Ashland, OR).

### Imaging

To enable identification of the different donor and receiving cell type in culture, the donor cell mitochondria were labeled with MitoTracker Red CMXRos (Molecular Probes, Invitrogen) and the CH (or half of the cells in CH → CH) were stained with CellTrace Violet (Invitrogen) according to the manufacturer’s instructions. To visualize tunneling nanotubes (TNTs), the donor cell type was stained with DiD (Vybrant™ Multicolor Cell-Labeling Kit; Invitrogen) in cocultures. In addition, the actin skeleton of all cells in all cultures was stained using 100 nM SiR-Actin (Spirochrome AG, Tebu Bio, Heerhugowaard, The Netherlands). Monolayers were imaged using a THUNDER fluorescence microscope and LASX acquisition software (both Leica microsystems, Wetzlar, Germany). TNTs were imaged using a Leica SP8X Laser Scanning Confocal Microscope (Leica microsystems) and LASX acquisition software.

### EV Isolation

To evaluate the presence of mitochondria in EVs and changes in EV secretion initiated by coculture, donor cells were dual-stained using CellTrace Violet and MitoTracker Red CMXRos as described in the “Quantification of Monolayer Mitochondrial Transfer” section or left unstained. Cells were cultured in monocultures or cocultures in 1:1 ratio for 24 hours in vesicle-deprived chondrocyte expansion medium, after which the conditioned medium was collected for processing. Cell debris were removed from conditioned medium by centrifugation for 5 minutes at 320*g*, followed by 15 minutes at 1,500*g*. Subsequently, the medium was centrifuged at 16,000*g* for 1 hour to pellet EVs.^31,32^ After discarding the supernatant, EVs were washed, resuspended in buffer, that is, PBS with 0.5% (w/v) bovine serum albumin (BSA; Roche Diagnostics GmbH, Mannheim, Germany) and 2 mM ethylenediaminetetraaceticacid (EDTA), and then analyzed using a BD LSRFortessa flow cytometer (BD Biosciences, Allschwil, Switzerland) and FlowJo V10 data analysis software package (Tree Star Inc). For each condition, 10,000 events were recorded.

### Delivery of MSC Mitochondria to CH in Monolayer

To investigate the effect of MSC-derived mitochondria on CH, mitochondria isolated from MSCs (prestained with MitoTracker Red CMXRos) were directly transferred into CH. MSCs were culture-expanded and half of the cells were treated with mitomycin C to induce senescence (sMSC). Mitochondria were isolated using the Mitochondria Isolation Kit for Cultured Cells (Thermo Scientific, Waltham, MA) according to the manufacturer’s instructions. Mitochondria were transferred into CH as previously described.^26^ Briefly, mitochondria were added to monolayers of CH and subjected to 2 consecutive centrifugation steps with an interval of 2 hours. The moment after the first centrifugation cycle was considered T0. Efficiency of MitoCeption on preseeded CH monolayers was measured using increasing concentrations of mitochondria. Then, isolated mitochondria of 9 × 10^5^ MSCs or sMSCs were used for MitoCeption on a monolayer of 1 × 10^5^ precultured CH to mimic a CH:MSC ratio of 10:90 as used in IMPACT.^6,33^ Intracellular location of the mitochondria was confirmed 1 day after MitoCeption with fluorescence microscopy and the effect of different dosages of mitochondria was assessed using flow cytometry. CH subjected to the same centrifugation steps without added mitochondria served as controls.

#### Metabolic activity of CH after mitochondrial transfer

Metabolic activity of the MitoCepted CH monolayers was determined directly after MitoCeption (T = 2 hours), after 26 hours, and after 44 hours using the conversion of resazurin to resorufin (44 mM; Alfa Aesar, Thermo Scientific) by measuring fluorescent intensity at 560 nm excitation and 590 nm emission.

#### Gene expression of CH after mitochondrial transfer

Total RNA of CH monolayers was isolated at T = 2 hours, T = 6 hours, T = 26 hours, and T = 46 hours after MitoCeption using TRIzol (Invitrogen) according to the manufacturer’s instructions. RNA was reverse-transcribed using the High-Capacity cDNA Reverse Transcription Kit (Applied Biosystems, Foster City, CA). Real-time polymerase chain reactions (PCRs) were performed using iTaq Universal SYBR Green Supermix (Bio-Rad) in the LightCycler 96 (Roche Diagnostics GmbH) according to the manufacturer’s instructions. Primers (Invitrogen) are listed in **Table 1**. Relative gene expression was calculated using 18S as a housekeeping gene and normalized for gene expression of that donor before MitoCeption. Amplified PCR fragments extended over at least 1 exon border (except for 18S).

**Table 1.** Primer Sequences for Quantitative Real-Time PCR.

Gene Name	Oligonucleotide Sequence (5′ to 3′)	Annealing Temperature (°C)	Product Size (bp)
18S	Fw: GTAACCCGTTGAACCCCATT Rv: CCATCCAATCGGTAGTAGCG	57	151
ACAN	Fw: CAACTACCCGGCCATCC Rv: GATGGCTCTGTAATGGAACAC	56	160
BCL2	Fw: GCGTCTGTAGAGGCTTCTGG Rv: GCCACTTGCCACTTTTCCTG	60	293
COL2A1	Fw: AGGGCCAGGATGTCCGGCA Rv: GGGTCCCAGGTTCTCCATCT	57	195

PCR = polymerase chain reactions; Fw = forward primer; Rv = reverse primer; ACAN = aggrecan; BCL2 = B-cell lymphoma 2; COL2A1 = collagen type II alpha 1 chain.

### Delivery of MSC Mitochondria to CH in 3D Chondropermissive Culture

To investigate whether transfer of MSC mitochondria into CH affects chondrogenesis, isolated mitochondria were transferred into CH during the pellet formation. Mitochondria of 9 × 10^5^ MSCs or sMSCs were isolated as described in “Direct mitochondrial transfer through MitoCeption in monolayer” and added to 1 × 10^5^ CH in suspension. Pellets of 1 × 10^5^CH were formed by centrifugation at 320*g* for 5 minutes in 15 ml Falcon tubes. MitoCeption on monolayers was performed in parallel to compare the efficiency of MitoCeption in pellets and in monolayers. Pellets were cultured for 28 days in chondropermissive medium, DMEM, 2% HSA, 2% (v/v) insulin-transferrin-selenium-ethanolamine (ITS-X; Gibco), 200 µM ASAP, and 1% pen/strep. Control pellets consisted of CH alone and CH:MSC cocultures in a 10:90 ratio (both 1 × 10^5^ total). Medium was changed twice per week and collected for analysis. After 1 and 2 weeks of culture, MitoCeption was repeated on a subset of pellets. Control pellets were also subjected to centrifugation at these time points. Results are displayed in Suppl. Fig. S2.

#### Release and deposition of glycosaminoglycans

Pellets were harvested after 28 days of culture and digested in a papain digestion buffer, 250 µg/ml papain (Sigma-Aldrich), 0.2 M NaH_2_PO_4_, 0.1M EDTA, 0.01M cysteine, pH 6.0, at 60 °C overnight. Deposition of sulfated GAG in the pellet digests and release into the culture medium was measured using a dimethylmethylene blue assay (DMMB; pH 3.0). Absorbance was measured at 525/595 nm using chondroitin-6-sulfate (Sigma-Aldrich) as a standard. DNA content of digests was quantified using Qubit dsDNA HS Assay Kit (Thermo Scientific) according to the manufacturer’s instructions.

#### Histological analyses

Pellets were processed for histology by fixation in a 4% buffered formaldehyde solution, followed by dehydration through graded ethanol steps, clearing in xylene, and embedding in paraffin. Sections of 5 µm were cut, stained with 0.125% safranin-O (Merck, Darmstadt, Germany), and counterstained with 0.4% fast green (Sigma-Aldrich) and Weigert’s hematoxylin (Clin-Tech, Glasgow, UK). Type I and II collagen deposition was visualized by immunohistochemistry. Sections were blocked in 0.3% (v/v) hydrogen peroxide, followed by antigen retrieval with 1 mg/ml pronase (Sigma-Aldrich) and 10 mg/ml hyaluronidase (Sigma-Aldrich), both for 30 minutes at 37 °C. Sections were blocked with 5% (w/v) BSA in PBS for 1 hour at room temperature and incubated with primary antibodies for type I collagen—EPR7785 (BioConnect, Huissen, The Netherlands), 1:400 in 5% PBS/BSA—and type II collagen—II-II6B3 (DHSB, Iowa City, IA), 1:100 in 5% PBS/BSA—overnight at 4 °C. For type I collagen, rabbit IgG (DAKO, Glostrup, Denmark; X0903) was used as isotype control, and for type II collagen, mouse IgG (DAKO X0931) was used. Next, type I collagen sections were incubated with BrightVision Poly-HRP-Anti Rabbit (VWR, Radnor, PA) and type II collagen sections were incubated with goat-anti-mouse IgG HRP-conjugated (DAKO, P0447; 1:100 in 5% PBS/BSA) for 1 hour at room temperature. Immunoreactivity was visualized using diaminobenzidine peroxidase substrate solution (DAB; Sigma-Aldrich). Mayer’s hematoxylin (Klinipath, Olen, Belgium) was used for counterstaining.

### DNA Analysis

DNA was isolated from digest of cartilage pellets at 0, 1, 2, and 4 weeks after culture and from the cartilage biopsies of 6 patients, taken 1 year after treatment with IMPACT ^6,7^ and from corresponding MSCs. Biopsies were compared with donor MSCs.

#### Extraction

DNA was extracted using the Qiamp DNA mini and blood mini kit according to the manufacturer’s protocol “DNA purification from blood or body fluids (spin protocol).” Hundred microliters of digested cells was added to 100 µl of PBS to acquire the appropriate volume. Elution was performed in 200 µl nuclease-free water.

#### Mitochondrial DNA analysis

A PCR was performed on 42 mitochondrial DNA SNPs (SNPs, primers, and input in primermix can be found in S3). PCRs were performed in a total volume of 12.5 µl with a mix containing 1.25 µl GeneAmp 10x PCR-bufferI (Applied Biosystems), 1.25 µl 10x dNTPs, 1.25 µl MT-DNA primermix, 1 µl MgCl_2_ (25 mM; Applied Biosystems), 0.5 µl Tag gold (5 U/µl; Applied Biosystems), and 7.25 µl of DNA extract. All PCRs were performed on a GeneAmp® PCR System 9700 using the following program: 94 °C for 10 minutes, 27 cycles of 94 °C for 30 seconds, 60 °C for 30 seconds, and 72 °C for 30 seconds and final extension of 5 minutes at 72 °C. A Qiaxcel run was performed to verify the amplification success. To prepare Illumina sequencing libraries from all PCR products, barcoded adapters were ligated to the PCR products using the KAPA library preparation kit® (KAPA Biosystems). An end-repair reaction was performed with 2.5 μl of PCR product in a total volume of 35 μl for 30 min at 20 °C. The A-tailing and adapter ligation were performed in a total volume of 25 μl for 30 min at 30 °C and 20 °C, respectively. For adapter ligation, barcoded adapters were used in a final concentration of 60 nM. During the preparation of libraries, no additional amplification occurred. Prepared libraries were quantified and subsequently pooled in equimolar amounts. Sequencing was performed on the MiSeq® Sequencer (Illumina) with 5% PhiX control library. Sequencing was performed according to the manufacturer’s protocol using v3 sequencing reagents. The MiSeq sequencing data were analyzed using a home-made pipeline that starts with fast length adjustment of short reads (FLASH),^34^ followed by TSSV^35^ and FDStools.^36^ FLASH was used to align paired-end reads and obtain a consensus sequence of higher quality. When paired-end reads differed more than 33%, they were discarded. When a difference less than 33% occurred between the 2 reads, bases with the highest quality score were incorporated in the consensus sequence. By providing TSSV with the primer sequences, the reads containing the region surrounding the SNP were recognized and counted. FDStools is a software tool package for the analysis of massive parallel sequencing data. It has the capability of recognizing and correcting noise from PCR or sequencing artifacts. FDStools was used to compare the sequence with the SNP to revised camREF, resulting in an HTML file for analyzation. All sequences with a percentage <5% of the most frequent sequence were filtered out. This cut-off is based on validation results that showed background noise is hardly ever higher than 5%. The MT-DNA results of the cultured samples were compared with the result of the MSC and CH MT-DNA to determine whether a mixture was present.

#### Autosomal DNA analysis

A VeriFiler™ Plus (Thermo Fisher Scientific) PCR was performed using 2.5 µl mastermix and 1.25 µl primermix in a total volume of 12.5 µl. The amount of input DNA was 0.5 ng, when possible. Otherwise 8.75 µl of DNA extract was added. PCR conditions were according to protocol. Capillary electrophoresis was performed on AB3500xL according to the manufacturer’s protocol. Results were analyzed using GeneMarker HID V2.9.5. The autosomal DNA results of the cultured samples were compared with the result of the MSC and CH autosomal DNA to determine whether a mixture was present.

### Statistical Analyses

**Figure 1.**
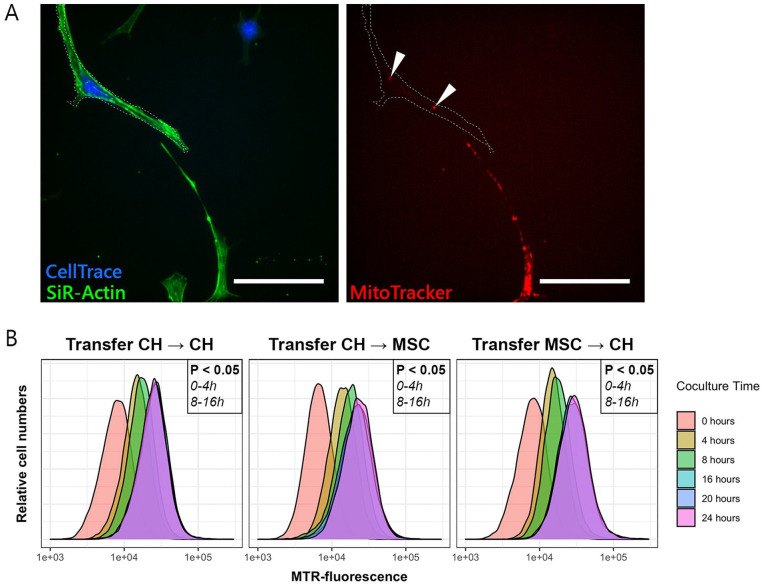
Transfer of mitochondria between chondrocytes (CH) and mesenchymal stromal cells (MSCs). (**A**) Stained mitochondria (MitoTracker, in red) are transferred from a donating MSC to a receiving CH stained with CellTrace (in blue). SiR-Actin stains F-actin in all cells (in green) and was used to visualize cell dimensions. Scale bar = 50 µm. (**B**) Quantification of transfer of mitochondria from donor to receiving cell, measured with flow cytometry. Mitochondrial transfer between all cell combinations (CH → CH, CH → MSC, and MSC → CH) occurred predominantly in the first 4 to 8 hours after initiation of the coculture. In all cases, 20,000 events were recorded. MTR = MitoTracker.

Data were analyzed using GraphPad Prism version 8.3.0 (GraphPad Software, San Diego, CA). Data are shown as mean ± standard deviation (SD) unless stated otherwise, and figure legends show the amount of donors and technical replicates (replicates of the same donor, cultured and measured separately). *P* values below 0.05 were considered statistically significant, and all tests were 2-sided. Analysis of variance (ANOVA) was used to test for significant differences in fluorescence between consecutive time-points (**Fig. 1**). As a follow-up, SIDAK correction for multiple comparisons was used. Two-way repeated-measures ANOVA was used to test for differences between senescent and inflammatory conditions and control condition (**Fig. 3**), CH control and MitoCeption groups (**Fig. 4**), and MitoCeption groups and CH/coculture controls (**Fig. 5**), taking into account donor variability. Here, a Dunnet’s *post hoc* test was performed to account for multiple comparisons. Normality of the data and homogeneity of variance were tested by visual expection of histograms and Q-Q plots. For gene-expression analysis, data of each donor were normalized for the average value in the control condition of that donor.

## Results

### Mitochondrial Transfer Takes Place Between CH and MSCs

All cells were stained with SiR-Actin to visualize cell dimensions. Cells stained with CellTrace (receiving cells) gained fluorescent mitochondria from donor cells that were stained with MitoTracker (**Fig. 1A**, indicated by white arrows). Stained mitochondria were transferred among CH, and between CH and MSCs. Using flow cytometry, mitochondrial transfer was quantified by measuring increase in fluorescence in receiving cells. Increase in fluorescence was significant from 0 to 4 hours and from 8 to 16 hours in all 3 coculture conditions. No further increase in fluorescence was found after 16 hours in any of the conditions (**Fig. 1B**).

### Mitochondrial Transfer Takes Place Through Direct Cell-Cell Contact, TNTs, and EVs

**Figure 2.**
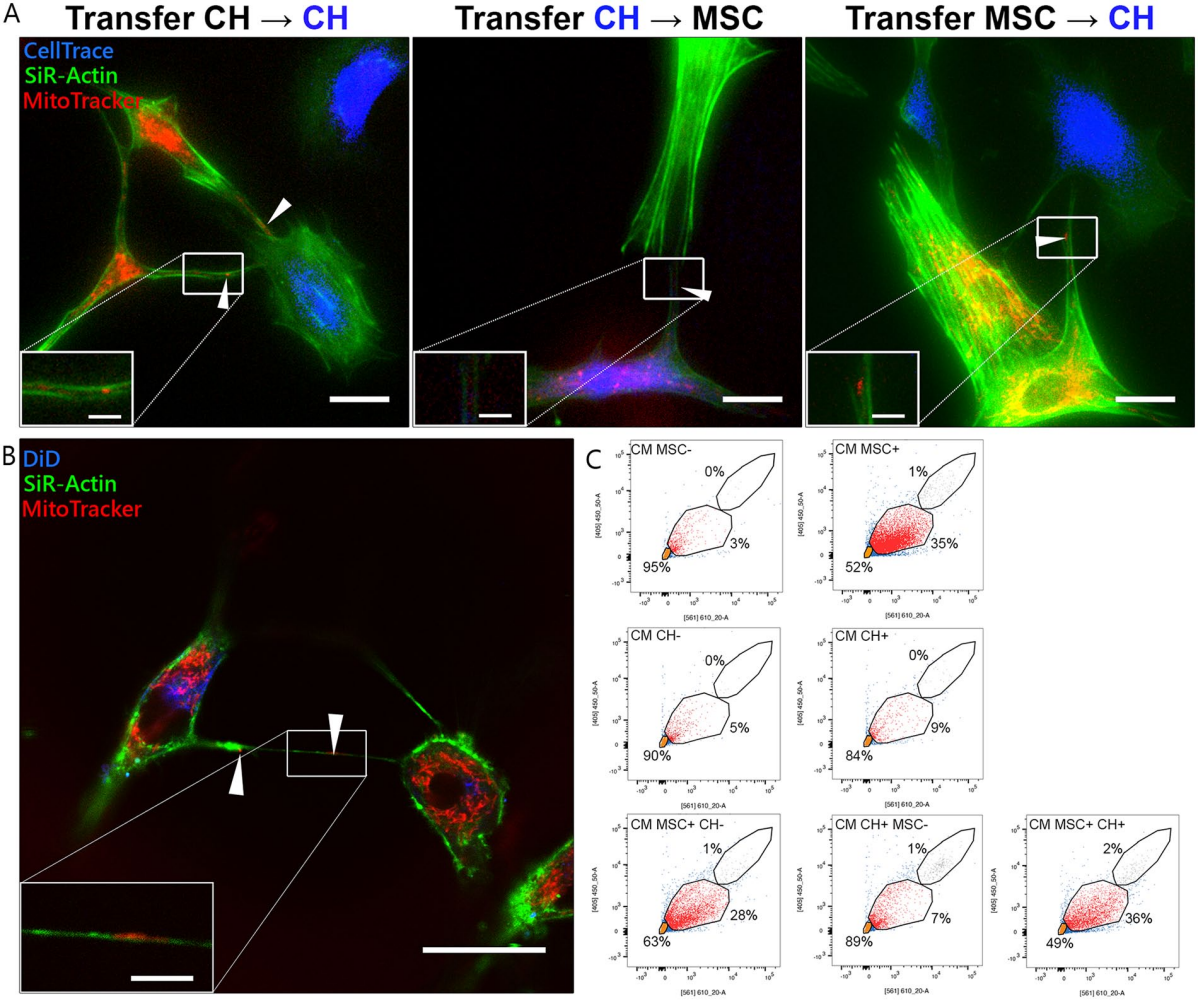
Bidirectional transfer of mitochondria between cells. (**A**) Visualization of mitochondrial transfer among chondrocytes (CH), between CH and mesenchymal stromal cells (MSCs), and vice versa after 7 hours of coculture. Donating cells were stained with MitoTracker (in red), CH were stained with CellTrace (in blue), and F-actin of all cells was stained with SiR-Actin (in green). Mitochondria transported between 2 cell types are indicated by white arrows. Scale bar = 25 µm. Time-lapse videos can be found in the supplemental information. (**B**) Transport of mitochondria through a tunneling nanotube between MSC and CH. Donating MSCs were stained with DiD (in blue) and MitoTracker (in red). F-actin of all cells was stained with SiR-Actin (in green). Image taken after 16 hours of coculture. Scale bar = 25 µm. (**C**) Flow cytometry analysis of (co)culture conditioned media (CM) for small particles including mitochondria-containing microvesicles (in red). Noise and particles negative for both dyes are depicted in orange, and cells (upper gate) are depicted in gray. Intensity of MitoTracker (561/610 nm) is depicted on the *x* axis, and intensity of CellTrace (405/450 nm) is depicted on the *y* axis. MSC− and CH− are unstained. MSC+ and CH+ are dual-stained for MitoTracker and CellTrace. In unidirectional cocultures (lower panels, left and middle), the first cell type is dual-stained, while the other is unstained. In the bidirectional coculture (lower panel, right), both cell types are dual-stained. In all cases, 10,000 events were recorded.

Transfer of mitochondria occurred through direct cell-cell contact (**Fig. 2A**), as mitochondria (MitoTracker, in red) were seen in broad actin-containing (SiR-Actin, in green) cell protrusions between 2 cell types (indicated by white arrows). In addition, transfer took place over larger distances as mitochondria were detected in TNTs between both cell types (**Fig. 2B**). A mitochondrion in a TNT is indicated by white arrow. Traces of DiD (in blue) are found in the receiving CH, suggesting transfer of the cytosolic dye from the stained MSCs. In conditioned medium of stained MSCs or CH, a population stained with MitoTracker as well as CellTrace (**Fig. 2C**, in red) was identified as EVs containing mitochondria. In conditioned medium of unstained cells, this population overlapped with the population identified as background noise (in orange). In conditioned medium of MSC monocultures, 35% of events were marked as mitochondria-containing EVs, whereas in conditioned medium of CH 9% of events were marked as mitochondria-containing EVs. In cocultures where only MSCs were stained, 28% of events were marked as mitochondria-containing EVs, whereas 7% of events were marked as mitochondria-containing EVs in the cocultures where only CH were stained. In cocultures where both CH and MSC were dual-stained, 36% of the events were mitochondria-containing EVs, suggesting that MSCs are stimulated to excrete EVs containing mitochondria in the presence of CH, while this is not the case for CH in the presence of MSC.

### Cell Stress Does Not Affect Mitochondrial Transfer

**Figure 3.**
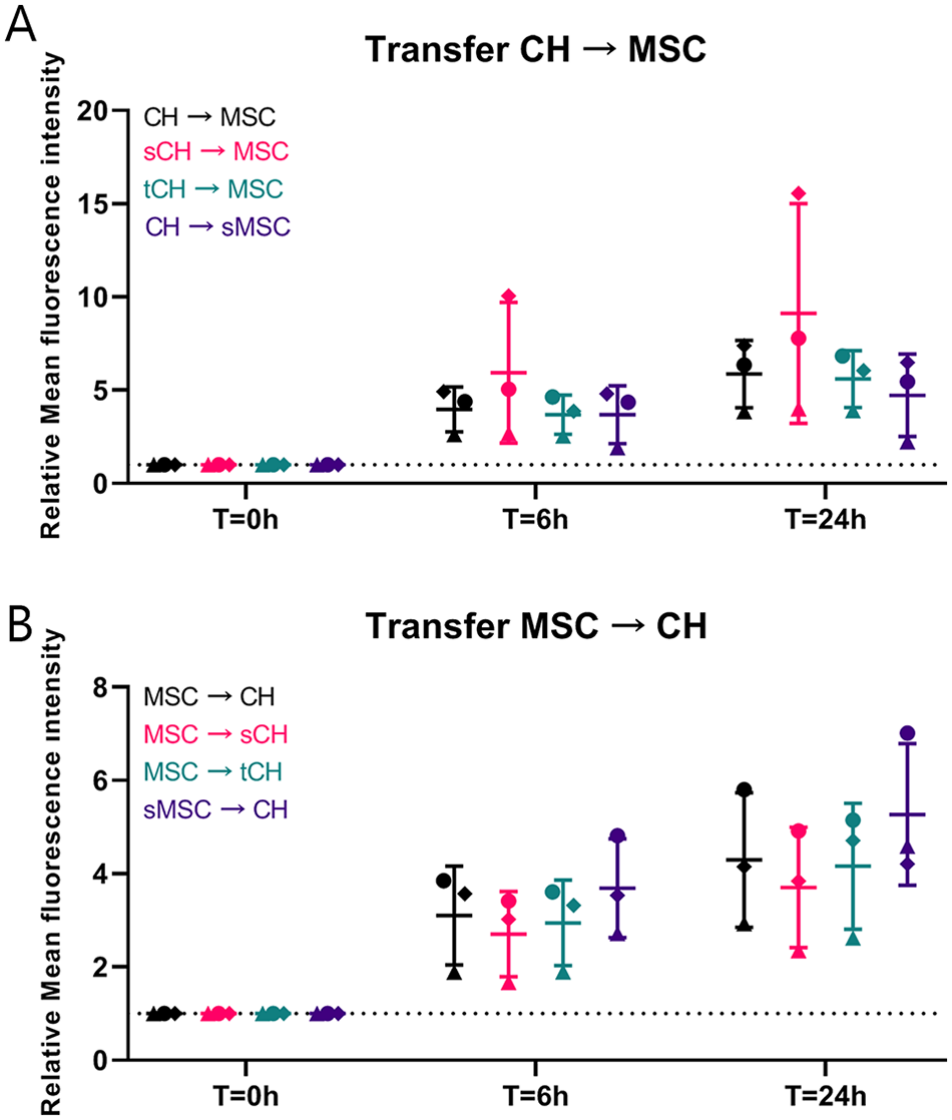
Effect of inflammation and senescence on mitochondrial transfer. (**A**) Mitochondrial transfer (measured using flow cytometry) from chondrocytes (CH), CH pretreated with tumor necrosis factor α (TNF-α) to induce inflammation (tCH), and CH pretreated with mitomycin C to induce senescence (sCH) to mesenchymal stromal cells (MSCs) and MSCs pretreated with mitomycin C to induce senescence (sMSC) tends to increase when CH are senescent (sCH). (**B**) Mitochondrial transfer (measured using flow cytometry) from sMSCs and MSC to CH tends to be increased in case of sMSC and sCH. Simulating an inflammatory environment using TNF-α in CH did not influence the speed and magnitude of mitochondrial transfer. Inflammation and senescence did not significantly change mitochondrial transfer. Error bars show standard deviations.

The effect of inflammation and senescence on mitochondrial transfer was investigated among CH and between CH and MSCs. Cells were pretreated with TNF-α or mitomycin C to mimic cell stress. There was no significant difference in transfer between any of the groups and the control condition, although over time the fluorescence intensity increased (**Fig. 3**).

### Uptake of MSC Mitochondria Increases Gene Expression of Aggrecan and B-Cell Lymphoma 2 in CH

**Figure 4.**
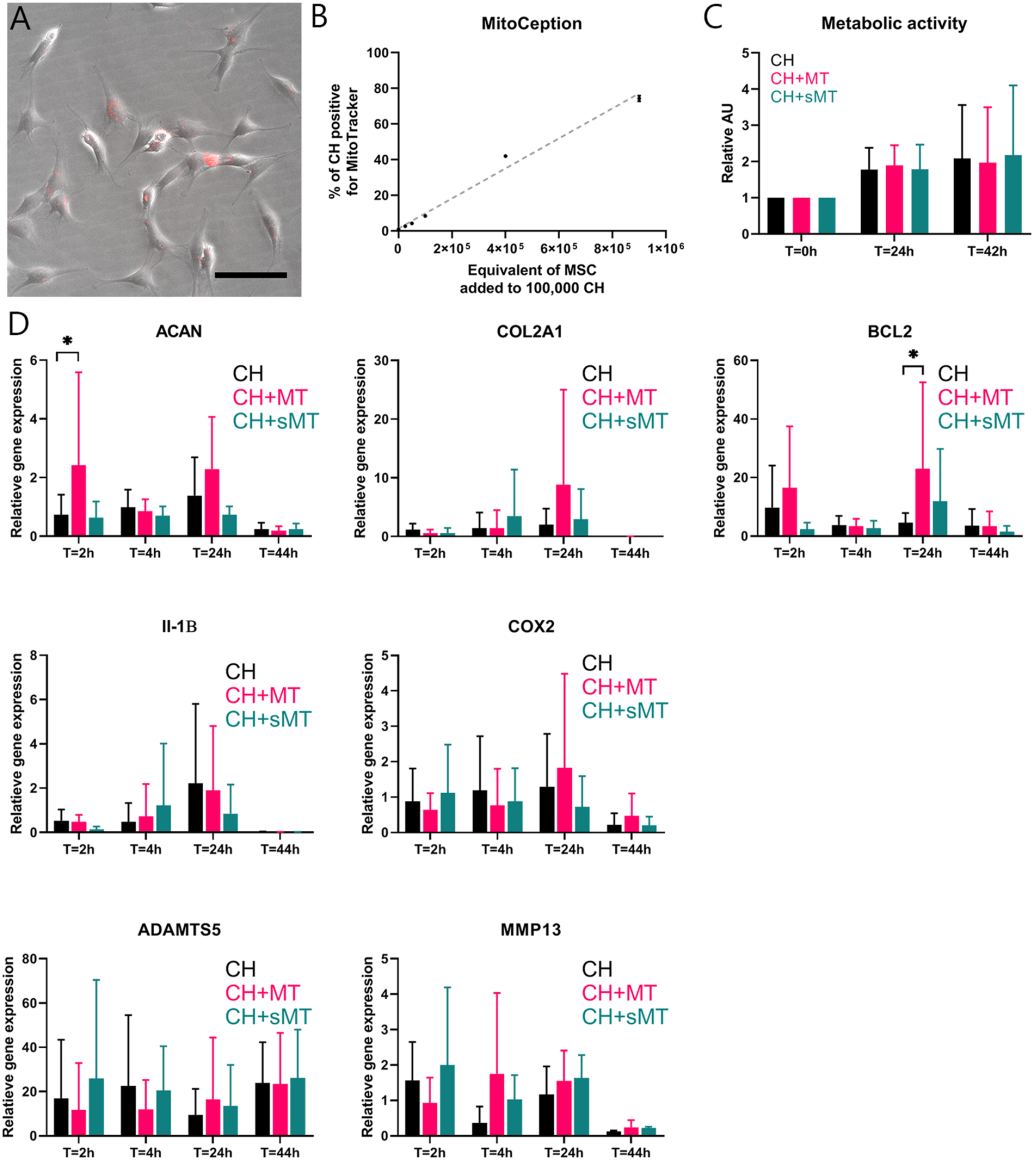
Direct mitochondrial transfer through MitoCeption. Mitochondria (MT) of 900,000 mesenchymal stromal cells (MSCs) were isolated and transferred into chondrocytes (CH) via MitoCeption. CH controls were subjected to the same centrifugation steps without the addition of MT. (**A**) MSC-derived MT, stained with MitoTracker (in red), localized intracellularly in CH monolayers. Scale bar = 100 µm. (**B**) Dose-dependent effect of MitoCeption using increasing concentrations of MT transferred into monolayers of 100,000 CH. Symbols depict averages of 2 measurements ± standard deviation, and the gray line shows linear regression. (**C**) Metabolic activity of CH monolayers as indicated by the conversion of resazurin to resorufin (ex: 560 nm, em: 590 nm) at 24 and 42 hours after MitoCeption with MT and senescent MT (sMT), both derived from 900,000 MSCs. *N* = 3 donor combinations. (**D**) mRNA expression of aggrecan (ACAN), type II collagen (COL2A1; both markers for chondrogenesis), and B-cell lymphoma 2 (BCL2; marker for cell survival) in CH monolayers at 2, 6, 26, and 46 hours after MitoCeption with MT and sMT derived from 900,000 MSCs. ACAN expression was increased in CH + MT compared with CH right after MitoCeption (T = 2 hours), and BCL2 was increased in CH + MT 26 hours after MitoCeption (T = 26 hours). *N* = 3 donor combinations, 2 technical replicates per donor. *P < 0.05. Error bars show standard deviations.

To assess the effects of MSC-derived mitochondria on CH in chondropermissive culture, mitochondria were transferred into CH by MitoCeption.^26,37^ Twenty-four hours after transfer, mitochondria (in red) were detected intracellularly in CH monolayers (**Fig. 4A**). The number of transferred mitochondria was dose-dependent as confirmed by flow cytometry (presented dose as equivalent to number of MSCs used for isolation). For further experiments, mitochondria of 900,000 MSCs were transferred onto 100,000 CH to mimic a cell ratio of 90:10, which is optimal for chondroinduction.^6,33^ When mitochondria of 900,000 MSCs were transferred on 100,000 CH, 74% ± 1.6% of the CH were positive for MitoTracker (**Fig. 2B**). Mitochondria derived from senescent MSCs were included to investigate whether these would exert similar effects as mitochondria from normal (proliferating) MSCs. MitoCeption of mitochondria and senescent mitochondria did not alter metabolic activity in CH monolayers at 24 and 42 hours of coculture (**Fig. 4C**). At T = 2 hours, mRNA expression of aggrecan (ACAN) was significantly upregulated in CH that received mitochondria compared with CH controls and CH that received senescent mitochondria. Expression of type II collagen (COL2A1) at 26 hours after MitoCeption with mitochondria was higher in 2 donor combinations, but not consistently among all donor combinations (*P* < 0.1). ACAN and COL2A1 expression declined at 46 hours in all groups. In addition, mRNA expression of B-cell lymphoma (BCL2), a marker for cell survival,^38^ was significantly higher 26 hours after MitoCeption with mitochondria, but not with senescent mitochondria (**Fig. 4D**). Individual values are shown in Suppl. Fig. S1.

### Transferred Mitochondria Exert a Chondrogenic Effect in Chondropermissive Culture

**Figure 5.**
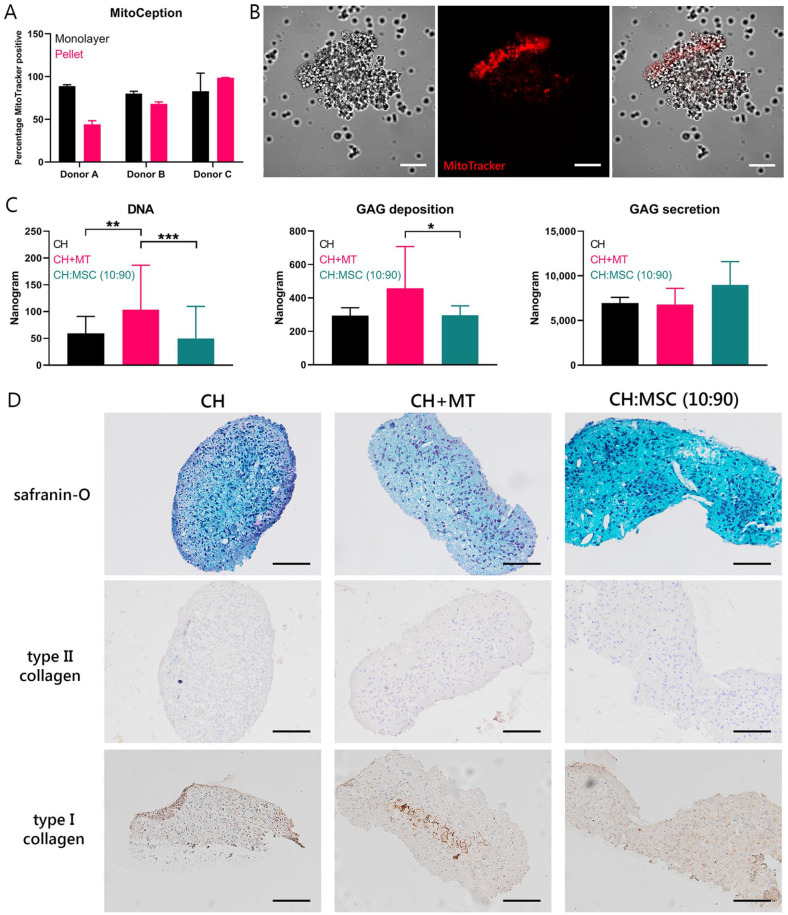
Chondrogenic effect of direct transfer of mitochondria. (**A**) Efficiency of transfer of MSC-derived mitochondria (MT) and senescent MSC-derived mitochondria (sMT) into chondrocyte (CH) pellets compared with MitoCeption on CH monolayers depicted for the 3 donor combinations. (**B**) Mitochondria stained with MitoTracker (in red) are localized in CH after simultaneous pelleting of cells and mitochondria. Scale bar = 100 µm. (**C**) Quantification of DNA and glycosaminoglycan (GAG) deposition and secretion of CH pellets after 28 days of chondropermissive culture in pellets. Control groups consisted of CH only and CH and MSC in coculture (CH:MSC, ratio 10:90). *P < 0.05, **P < 0.01, ***P < 0.001. (**D**) Histological analysis for proteoglycans (safranin-O), type II collagen, and type I collagen. Scale bar = 100 µm. Error bars show standard deviations.

To investigate the effect of transferred mitochondria on cartilage EV production *in vitro*, isolated mitochondria from MSCs were transferred into CH using MitoCeption during formation of cell pellets at initiation of the culture. Efficiency of the MitoCeption protocol in pellets was compared with the efficiency of monolayers (**Fig. 5A**). Efficiency in pellets was comparable to monolayers in 2 donors and lower in 1 donor (donor A, 89% ± 1.5% vs. 44% ± 4.4%). Transferred mitochondria (in red) are detected in CH pellets 1 day after initiation of the culture (**Fig. 5B**). Brightness of MitoTracker was higher in one side of the pellet, where more cells were stacked on top of each other. Stained mitochondria were found throughout the entire pellet. After 28 days of chondropermissive culture, the amount of DNA was higher in pellets that received mitochondria (CH + MT) compared with control CH pellets (CH) and CH and MSC cocultures (CH:MSC [10:90]) (**Fig. 5C**, left panel). Similarly, the amount of GAGs deposited in the pellets was higher compared with the CH and MSC coculture and showed a similar trend (*P* < 0.1) compared with CH (**Fig. 5C**, middle panel). Secretion of GAGs into the culture medium was not different between the 3 groups (**Fig. 5C**, right panel). GAG deposition was insufficient to result in positive safranin-O staining in all groups. The type II collagen staining was negative in all pellets. There was a slight staining positive for type I collagen, especially in the center of the CH with MSC mitochondria pellet (**Fig. 5D**). When mitochondria were transferred to CH pellets using MitoCeption after 1 or 2 weeks of culture, mitochondria were taken up only in the surface of the pellets and did not reach the inner CH (Suppl. Fig. S2).

### Mitochondrial and Autosomal DNA Quantification

**Figure 6.**
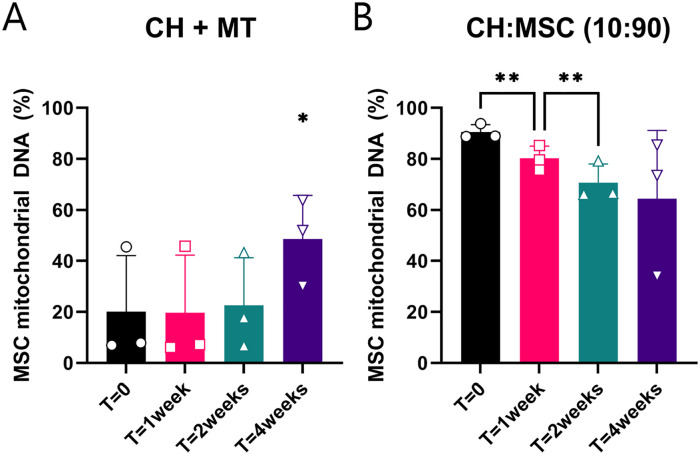
Mitochondrial DNA from mesenchymal stromal cell (MSC) donors present in cultured pellets. (**A**) Chondrocytes (CH) cultured with mitochondria (MT) of MSCs. (**B**) Chondrocyte and MSC cocultures in 10:90 ratio. *P < 0.05, **P < 0.001. Error bars show standard deviations.

Using a mitochondrial DNA SNP assay, 42 amplicons of the mitochondrial DNA were analyzed to assess the contribution of CH and MSC DNA in pellets after 0, 1, 2, and 4 weeks of culture. In the CH that received mitochondria, the relative amount of mitochondrial MSC DNA increased between 0 and 4 weeks. In the CH and MSC cocultures, the relative amount of mitochondrial MSC DNA decreased from 0 to 1 week and from 1 to 2 weeks (**Fig. 6**). At 4 weeks, the mitochondrial MSC DNA was 64% of the total DNA, whereas the autosomal DNA of the CH was the highest contribution at this time point (data not quantifiable).

The mitochondrial DNA of cartilage biopsies of 6 patients, taken 1 year after treatment with 90% allogeneic MSCs and 10% autologous chondrons, was also analyzed for the presence of mitochondrial DNA of the MSC donors. No donor mitochondrial DNA could be detected in the biopsies. Absence of autosomal MSC DNA was already determined before.

## Discussion

In this study, we demonstrated bidirectional transport of mitochondria between CH and MSCs for the first time. In addition, we identified 3 mechanisms responsible for mitochondrial transport, which are direct cell-cell contact, TNTs, and EVs. Finally, we showed compelling evidence of a chondrogenic effect of transferring MSC-derived mitochondria to CH through MitoCeption, indicating that mitochondrial transfer might be one of the underlying mechanisms of MSC-induced chondrogenesis.

Mitochondrial transfer could have an important role in the prevention or treatment of this mitochondrial dysfunction. Transfer of mitochondria is initiated in the first hours of coculture and reaches an equilibrium after 16 hours. The timing of mitochondrial transfer was not explicitly researched before, but others have found indications of mitochondrial transfer at 10 to 12 hours from MSCs to CH^18,19^ and at 4 hours between MSCs and macrophages.^15^ Interestingly, the transport of mitochondria occurs not only from MSCs to CH, but CH also transfer mitochondria to MSCs. Different explanations of this transfer could be hypothesized. The transfer of defective mitochondria from CH toward MSCs might be a damage signal, as transfer by cardiomyocytes and endothelial cells induced the antiapoptotic function of MSCs and secretion of cytoprotective enzymes.^39^ Moreover, defective mitochondria could be excreted by CH for degradation by MSCs, a process known as transmitophagy.^40^ Finally, depolarized mitochondria might be recycled by fusion with recipient cell mitochondria, increasing the metabolic state of the recipient.^41^ To summarize, uptake of healthy MSC mitochondria by CH would benefit the metabolic state, while clearance of defective mitochondria could prevent the damage caused by oxidative stress.

Direct cell-cell contact, TNTs, and EVs are all mechanisms for mitochondrial transfer. The importance of direct cell-cell contact between MSCs and CH for *in vitro* chondroinduction has been shown earlier.^12^ In direct cocultures, expression of gap junction protein connexin 43 was upregulated.^12^ Although mitochondria cannot physically pass gap junctions, connexin 43 is a mediator of mitochondrial transport.^42^ In fact, connexin 43 was reported to be essential in EV-mediated mitochondrial transfer between MSCs and alveolar cells.^43^ MSC-derived EVs enhance chondrogenesis of osteoarthritic CH *in vitro*.^44^ Here, mitochondria containing EVs were identified, indicating that mitochondria might play a role in the chondrogenic effect of MSC-derived EVs. Mitochondrial transport through TNT is another frequent mechanism for transport of mitochondria,^45^ and it has been described to occur between human MSCs and renal tubular cells,^46^ cardiomyocytes,^47^ vascular smooth muscle cells,^48^ and endothelial cells.^49^ TNTs likely play a pivotal role in the transport between MSCs and CH, which is shown for the first time in the current study. Next to mitochondrial transfer, TNTs allow transfer of various cellular components, including proteins, lysosomes, and RNA,^45^ which was not studied here but could provide other explanations of the MSC-CH coculture mechanism.

Upon addition of MSC mitochondria to CH, DNA content and proteoglycan deposition increased; thus, mitochondrial transfer might play an important role in the chondrogenic effect of MSCs. Gene expression showed increased ACAN and BCL2 expression, indicating a possible chondroinductive effect as well as increased survival. Similarly, a higher expression of type II collagen and proteoglycans was described^20^ in osteoarthritic CH that had taken up MSC mitochondria. In the current study, an increase in type II collagen deposition could not be demonstrated with immunohistochemistry. Overall, the deposition of type I and II collagen was low, and the GAGs present after pellet culture were not abundant enough to result in red safranin-O staining. This could be attributed to the fact that no growth factors were added in the chondropermissive culture. In the study by Wang *et al.*,^20^ increased chondrogenesis might be attributed to EVs or trophic factors as well, as it was studied in coculture. The increased chondrogenesis in CH with MSC mitochondria might be at least partially explained by promoting cell survival or proliferation in CH by restoring the energy balance,^37^ because matrix production per cell did not increase in chondropermissive cultures. Another effect of mitochondrial transfer might be the regulation of autophagy^50^ because autophagy is activated under hypoxic stress conditions^51^ and protects against mitochondrial dysfunction. This interaction could be the focus of follow-up research.

*In vitro*, the contribution of DNA of transferred MSC mitochondria increased between 2 and 4 weeks, indicating that there is a sort of selective advantage of MSC mitochondria above CH mitochondria in culture. Moreover, the contribution of MSC mitochondrial DNA in cocultures exceeded the contribution of MSC autosomal DNA in these cultures. This could indicate that this positive selection for MSC mitochondria also takes place in cocultures. However, the fate of transferred mitochondria and the occurrence of mitochondrial transfer *in vivo* remain unknown, as we could not detect mitochondrial DNA of donor MSCs in cartilage biopsies taken 1 year after cell therapy with autologous chondrons and allogeneic MSCs. Earlier studies have shown the presence of human mitochondrial DNA up to 28 days in murine macrophages.^41^ Similarly, the autosomal MSC DNA decreases in 28 days of coculture,^12^ and no autosomal MSC DNA can be detected *in vivo* after 1 year.^6^ The possibility that mitochondrial transfer occurs solely *in vitro* cannot be excluded, but mitochondrial transfer has been shown between MSCs and cardiac^39^ or alveolar cells^43^ *in vivo.* More likely, donor mitochondria are not retained in receiving CH over a prolonged period.

### Limitations

In contrast to our hypothesis, mimicking cell stress conditions using induction of inflammation or senescence did not significantly alter mitochondrial transfer. Similarly, inflammation induction by interleukin-1β treatment did not alter total transfer during 10 hours of coculture of CH and MSCs as described by Bennett *et al*.^19^ Inflammation might not play an important role in mitochondrial transfer, or the inflammatory phenotype resulting from these treatments is not well retained *in vitro* after removing the factors. Similarly, senescence did not change mitochondrial transfer significantly. *In vivo*, senescence is induced by mechanical stress in the rim of cartilage defects^52^ and drives aging and related pathologies. In osteoarthritis, senescent cells excrete catabolic factors causing cartilage degradation. Here, senescence induction by mitomycin C did not alter total mitochondrial transport. Senescence and the resulting formation of reactive oxygen species (ROS) might compromise the quality and number of mitochondria, but this was not investigated here. The generalizations of this study are limited by the *in vitro* character of the experiments. However, primary human cartilage defect CH were used together with MSCs from our Good Manufacturing Practices (GMP)-certified cell therapy facility to closely mimic the clinical situation and allow to test all groups and conditions using 3 donor combinations.

### Implications

The presented results demonstrate the role of mitochondrial transport in the chondroinductive effect of MSCs on CH. Treatment with MSCs or mitochondria in the acute phase of cartilage injury might prevent or treat mitochondrial dysfunction and subsequent ROS accumulation, and therefore counteract one of the first steps toward development of osteoarthritis.^24^ Moreover, preselection of MSCs for their capacity to donate functional mitochondria or take up damaged mitochondria for degradation could enhance the effect of MSCs in cocultures. Eventually, the potential of MSC-derived mitochondria as a method for cell-free therapies could be explored. Cell-free therapies have advantages including lower safety profiles and homogenization of treatment. However, limiting treatment to mitochondria disregards other possible functions of MSCs such as transmitophagy of defective mitochondria and reactivity to damage signals with trophic factors, EVs, or TNT communication. In addition, efficient long-term storage of mitochondria should be investigated and chondroinductive potency upon thawing should be confirmed.^53^

## Supplemental Material

sj-docx-1-car-10.1177_19476035221126346 – Supplemental material for Mitochondrial Transport from Mesenchymal Stromal Cells to Chondrocytes Increases DNA Content and Proteoglycan Deposition In Vitro in 3D Cultures Click here for additional data file. Supplemental material, sj-docx-1-car-10.1177_19476035221126346 for Mitochondrial Transport from Mesenchymal Stromal Cells to Chondrocytes Increases DNA Content and Proteoglycan Deposition In Vitro in 3D Cultures by Jasmijn V. Korpershoek, Margot Rikkers, Fleur S. A. Wallis, Koen Dijkstra, Marije te Raa, Peter de Knijff, Daniel B. F. Saris and Lucienne A. Vonk in CARTILAGE
