# Supplementary material for: Mitochondrial Transport from Mesenchymal Stromal Cells to Chondrocytes Increases DNA Content and Proteoglycan Deposition In Vitro in 3D Cultures
Source: Cartilage. 2022 Oct 19;13(4):133–47. doi: 10.1177/19476035221126346 (PMC9924973; doi:10.1177/19476035221126346)
Supplement: sj-docx-1-car-10.1177_19476035221126346 – Supplemental material for Mitochondrial Transport from Mesenchymal Stromal Cells to Chondrocytes Increases DNA Content and Proteoglycan Deposition In Vitro in 3D Cultures [file sj-docx-1-car-10.1177_19476035221126346.docx]

**Supplemental videos. Bidirectional transfer of mitochondria between cells.** Visualization of mitochondrial transfer among chondrocytes (CH) and CH, between CH and mesenchymal stromal cells (MSC), and vice-versa after 7 hours of co-culture. Donating cells were stained with MitoTracker (in red), CH were stained with CellTrace (in blue), and F-actin of all cells was stained with SiR-Actin (in green). Images taken at 40X magnification with 2 minute intervals.

# Supplemental Information

**Figure S1. Direct mitochondrial transfer through MitoCeption**. Mitochondria (MT) of 900,000 mesenchymal stromal cells (MSCs) were isolated and transferred into chondrocytes (CH) via MitoCeption. mRNA expression of aggrecan (ACAN), type II collagen (COL2A1; both markers for chondrogenesis), and B-cell lymphoma 2 (BCL2; marker for cell survival) in chondrocyte monolayers at 2, 6, 26, and 46 hours after MitoCeption with MT and sMT derived from 900,000 MSCs. ACAN expression was increased in CH+MT compared to CH right after MitoCeption (T=2h), BCL2 was increased in CH+MT 26 hours after MitoCeption (T=26h). N = 3 donor combinations, 2 technical replicates per donor. **p*<0.05


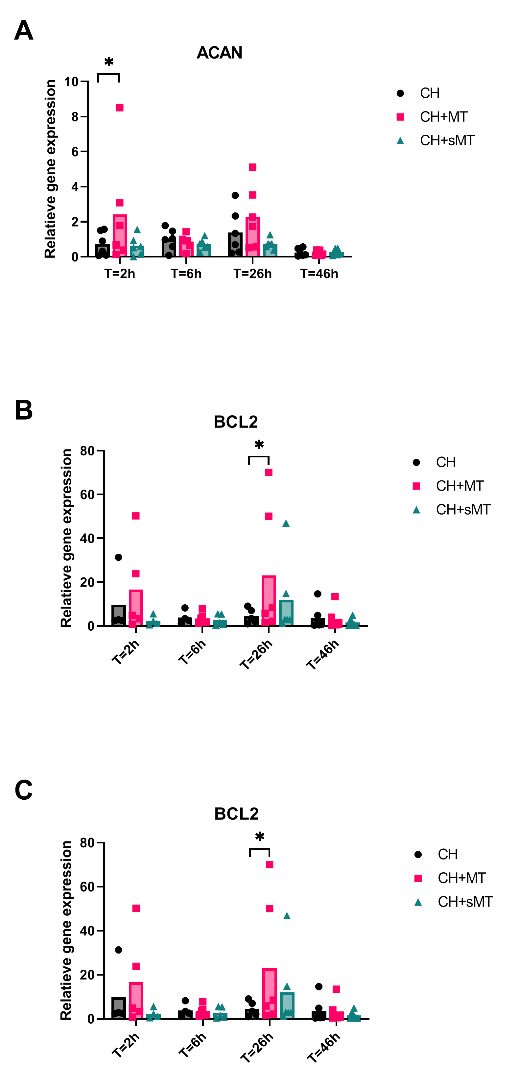

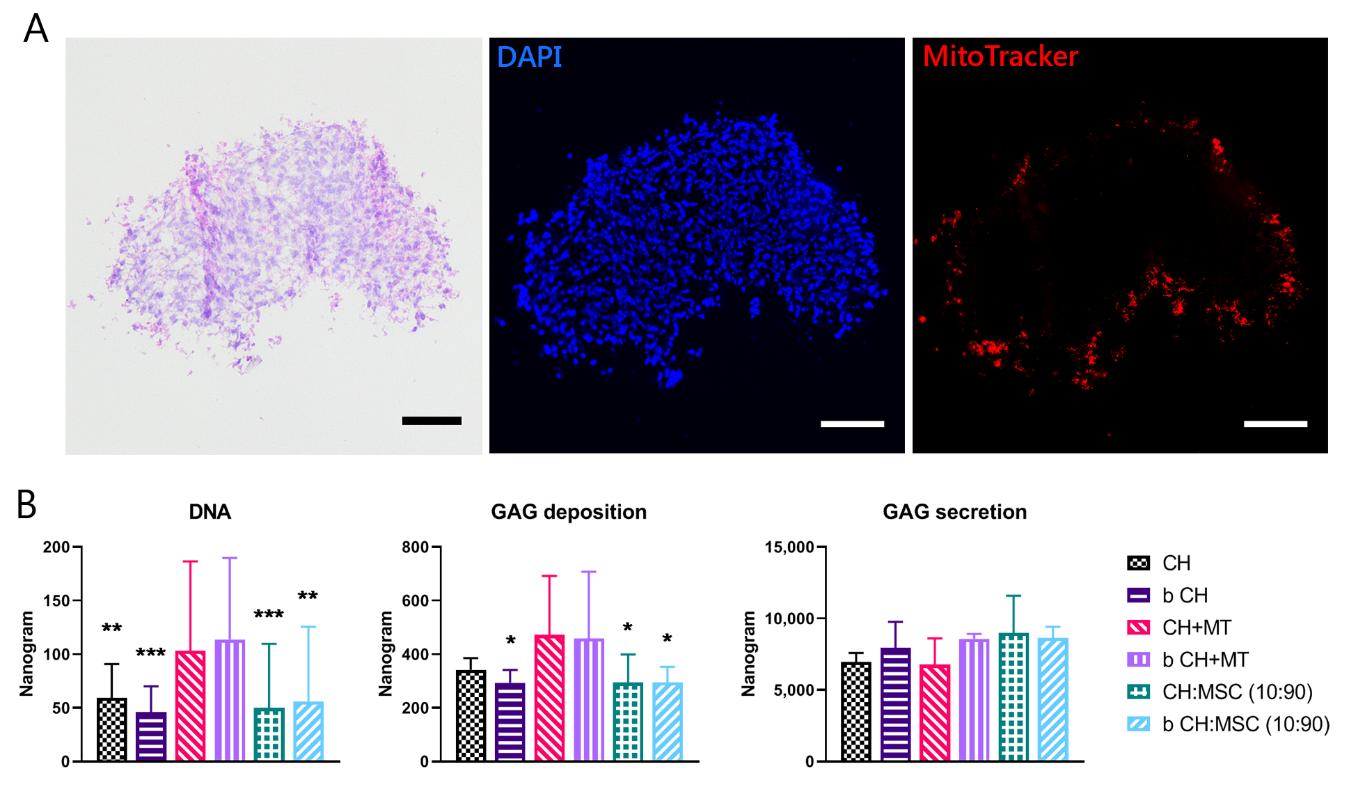


**Figure S2. Chondrogenic effect of periodically repeating MitoCeption.** A subset of chondrocyte (CH) pellets received additional doses of mitochondria (MT) at 7 and 14 days during chondropermissive culture. (A) Haematoxylin and eosin staining of re-MitoCepted pellet at 14 days (left panel), 4’,6-diamidino-2-phenylindole (DAPI, middle panel), and MitoTracker (right panel) of the same pellet show detection of transferred mitochondria in the perimeter of the pellet. Scale bar = 100 µm. (B) Quantification of DNA and glycosaminoglycan (GAG) deposition and secretion of CH pellets after 28 days of chondropermissive culture in pellets. Group bCH+MT received additional doses of mitochondria (boost) at 7 and 14 days in culture. Additional control groups bCH and bCH:MSC (10:90) were also subjected to centrifugation at these time points. **p*<0.05, ***p*<0.01, ****p*<0.001.

**S3 SNP’s, primers and input in primermix.**

| **Marker** | **Forward primer** | **Reverse Primer** | **µl (f+r mix 50pmol/µL)** |
| --- | --- | --- | --- |
| mt239(234-247) | tgtaaaacgacggccagtGTGTTAATTAATTAATGCTTgTrrGACAT | caggaaacagctatgaccGAAAGTGGcTGTGCAGACrTT | 15,6 |
| mt456+462+477(455-478) | tgtaaaacgacggccagtcACCCCcCAACTAACACATTATTT | caggaaacagctatgacccGgGGgTTGTaTTGrTGrGAT | 14,4 |
| mt930(927-931) | tgtaaaacgacggccagtCACACGATTAACCCAAGyCAATA | caggaaacagctatgaccTGATcTAAAACACTCTTTACGCCG | 4,4 |
| mt1018(1017-1031) | tgtaaaacgacggccagtAAAAACTCCAGtTGACACAAAAT | caggaaacagctatgaccGCTATTGTGTgTTCAGAtATGTTAAAG | 4,4 |
| mt1189(1188-1194) | tgtaaaacgacggccagtGACCTGGCGGTGCTTCAT | caggaaacagctatgaccCGATTACAGAACAGGCTCCTCTA | 5,0 |
| mt1438(1431-1446) | tgtaaaacgacggccagtGGTCGAAGGTGGATTTAGCA | caggaaacagctatgaccGGCCcTGTTCAACTAAGCAC | 4,1 |
| mt1738(1734-1748) | tgtaaaacgacggccagtAACCTTAgCcAAACCATTTACC | caggaaacagctatgaccCGCCAGGTTTCAATTTCTATCG | 3,8 |
| mt2706(2698-2708) | tgtaaaacgacggccagtATTGACCTGCCCGTGAAGAG | caggaaacagctatgaccGGGTCTTCTCGTCTTGCTGT | 7,5 |
| mt3010(3006-3013) | tgtaaaacgacggccagtCTCGATGTTGGATCAGGACA | caggaaacagctatgaccACCTTTAATAGCGGCTGCAC | 5,6 |
| mt3333(3330-3337) | tgtaaaacgacggccagtCCATGgCCAACCTCCTACT | caggaaacagctatgaccCCATTGCGATtAGAATGGGTA | 6,5 |
| mt3423(3421-3441) | tgtaaaacgacggccagtCTACGCAAAGGCCCCAAC | caggaaacagctatgaccCGTCAGCGAAgGGyTGTAG | 15,0 |
| mt3516(3512-3519) | tgtaaaacgacggccagtAAACCCgccACATCTrCCATCA | caggaaacagctatgaccAAGGTcGGGGCGGTGAT | 6,3 |
| mt3796(3796-3801) | tgtaaaacgacggccagtAGTGGCTCCTTTAACCTCTCC | caggaaacagctatgaccGAGGTGTTCTTGTGTTGTGAT | 6,3 |
| mt4793(4792-4795) | tgtaaaacgacggccagtCATAaTaGCTATAGCAATAAAACTAGGAA | caggaaacagctatgaccGGGACTCAGAAGTGAAAGGGG | 5,0 |
| mt5004(4998-5010) | tgtaaaacgacggccagtTAAACCAAACCCArCTACGC | caggaaacagctatgaccCCTATgTGGGTAATTGAGGAGT | 3,8 |
| mt6371(6364-6376) | tgtaaaacgacggccagtTCTTCTCCTTACACCTAGCAG | caggaaacagctatgaccTGATGAAaTTGATGGCCCCTAAG | 4,4 |
| mt6776(6771-6785) | tgtaaaacgacggccagtCCTaGGgTTTATCGTGTGAGCA | caggaaacagctatgaccCGTGTGTCTACGTCTATTCCTAC | 3,5 |
| mt7768(7762-7780) | tgtaaaacgacggccagtATACTAACATCTCAGACGCTCA | caggaaacagctatgaccTGATGGCGGGcAGGATAGT | 3,8 |
| mt8448(8445-8460) | tgtaaaacgacggccagtACTATTcCTCATCACCCAACTAA | caggaaacagctatgaccGTGAGGGaGGTAgGTGGTAG | 6,9 |
| mt8697(8694-8705) | tgtaaaacgacggccagtTGACTAATCAAACTAAcCTCAAAACA | caggaaacagctatgaccGGTTCGTCCTTTAGTGTTGTGT | 3,8 |
| mt9716(9715-9724) | tgtaaaacgacggccagtGCACTGCTtATTACAATTTTACTGG | caggaaacagctatgaccTCTGAGGCTTGTAGGAGGGTA | 3,8 |
| mt10034(10028-10035) | tgtaaaacgacggccagtGTATAAATAGTACCGTTAACTTCCAATT | caggaaacagctatgaccGTTTATTACTCTTTTTTGAAtGTTGTCAA | 4,4 |
| mt10211(10198-10212) | tgtaaaacgacggccagtCTTcGACCCTATATyCCCCG | caggaaacagctatgaccGGTaATAGCTACTAAGAAGAATTTTATGG | 5,0 |
| mt10873(10872-10876) | tgtaaaacgacggccagtCCACAGCCTAATTATTAGCATCATCC | caggaaacagctatgaccAGGTTGtTGTtGATTTGGTTAAAAAATAG | 4,4 |
| mt11176(11164-11186) | tgtaaaacgacggccagtACcTTggcTATCATCACCCG | caggaaacagctatgaccGAaGTATGTgCCTGCGTTCA | 3,1 |
| mt11251(11250-11260) | tgtaaaacgacggccagtCCTTCCCCTACTcATCGCAC | caggaaacagctatgaccAGTGAGCCTAGgGTGTTGTG | 5,0 |
| mt11332(11330-11340) | tgtaaaacgacggccagtCCCAAGAaCTATCAAACTCCTGA | caggaaacagctatgaccAAGCTATTGTGTAaGCTAGTCATATT | 3,8 |
| mt11467+11485(11466-11490) | tgtaaaacgacggccagtAGTACTTGCCGCAGTACTCT | caggaaacagctatgaccTGAGAATGAGTGTGAGGCGT | 4,4 |
| mt11719(11709-11725) | tgtaaaacgacggccagtCGGCGCAgTCATtCTCATAA | caggaaacagctatgaccGCTAGGCAGAATAGTArTGAGGA | 4,4 |
| mt11812(11810-11830) | tgtaaaacgacggccagtTCCTCTCTCAAGGaCTTCAAACT | caggaaacagctatgaccAGGCTTGCTAgAAGTCATCA | 4,4 |
| mt11947(11941-11951) | tgtaaaacgacggccagtTCTCCTGATCAAaTATCACTCTCCT | caggaaacagctatgaccAGGGcTGTGAcTAGtATGTTGA | 3,8 |
| mt12633(12629-12641) | tgtaaaacgacggccagtCCCtGTAGCATTgTTCGTTACAT | caggaaacagctatgaccGgTCTGAGTTTATATATCACAGTGAGAAT | 3,8 |
| mt12705(12693-12705) | tgtaaaacgacggccagtGAcCCAAACATTAATCAGTTCTTCAA | caggaaacagctatgaccGTaACTAAGATTAGtATGGTaATTAGGAA | 5,0 |
| mt13617(13613-13623) | tgtaaaacgacggccagtAAGCGCCTATAGCACTCGAA | caggaaacagctatgaccCGAGGTtGaCCTGTTAGGGT | 5,6 |
| mt13789(13789-13791) | tgtaaaacgacggccagtCTTCCAAACAACAaTCCCCCTC | caggaaacagctatgaccGCGAGGGCtGTGAGTTTTAG | 7,5 |
| mt14470(14461-14471) | tgtaaaacgacggccagtTACTCCTCAATAGCCATCGC | caggaaacagctatgaccGGGGaATGaTGGTTGTyTTTG | 5,3 |
| mt14766(14764-14788) | tgtaaaacgacggccagtCCAATGACCCCaATACGCAA | caggaaacagctatgaccAGGTCGATGAaTGAGyGGTT | 15,6 |
| mt14783+14793+14798(14780-14801) | tgtaaaacgacggccagtACGCAAAAcTAaCCCCCTAATA | caggaaacagctatgaccATGGGGTGGGGAGGTCGA | 11,3 |
| mt15257(15252-15262) | tgtaaaacgacggccagtTCAATGaaTCTGAGGaGGCTACT | caggaaacagctatgaccAAGAATCGTGTGAGGGTGGG | 4,4 |
| mt15775(15774-15776) | tgtaaaacgacggccagtCTGAaTCGGAGGaCAACCAG | caggaaacagctatgaccCCAATGATGGTAAAAGGGTAGC | 6,6 |
| mt15904+15907(15896-15908) | tgtaaaacgacggccagtTCAAATGggCCTGTCCTTGT | caggaaacagctatgaccTccGGtTTACAAGACTGGTGT | 4,3 |
| mt16162(16153-16176) | tgtaaaacgacggccagtCgGTACCAtAAAtACTtgrCyACCT | caggaaacagctatgaccATGGGGAGGGGGTkTtGAT | 8,8 |
| Nuclease free water | - | - | 58,6 |
